# Parental psychological distress in the postnatal period in Japan: a population-based analysis of a national cross-sectional survey

**DOI:** 10.1038/s41598-020-70727-2

**Published:** 2020-08-13

**Authors:** Kenji Takehara, Maiko Suto, Tsuguhiko Kato

**Affiliations:** 1 Department of Health Policy, National Center for Child Health and Development, 10-1, Okura 2 chome, Setagaya, Tokyo, 157-8535 Japan; 2 Department of Social Medicine, National Center for Child Health and Development, 10-1, Okura 2 chome, Setagaya, Tokyo, 157-8535 Japan

**Keywords:** Epidemiology, Risk factors

## Abstract

Mental health assessments of both members of a couple are important when considering the child-rearing environment. The prevalence and factors associated with both parents’ psychological distress have not been fully investigated. A nationally representative sample from the 2016 Comprehensive Survey of Living Conditions in Japan was used to examine the prevalence of moderate and severe psychological distress in parents in the first year after childbirth. In total, 3,514 two-parent households raising children under one year old met the study criteria. The Japanese version of Kessler 6 was used to assess moderate and severe psychological distress. The prevalence of either or both parents experiencing psychological distress in the first year after birth were 15.1% and 3.4%, respectively. A multivariate logistic regression analysis showed factors of fathers who worked ≥ 55 h a week, reduced duration of sleep in mothers, age in months of the youngest child, and high household expenditures were significantly associated with both parents simultaneously having moderate or severe psychological distress. This study implied the importance of prevention and early detection of parental psychological distress in both parents. Assessing parents’ psychological distress and work-style reform in the childcare period is an urgent issue to improve their mental health conditions.

## Introduction

Psychological distress in the postpartum period often manifests as depressive episodes in both men and women and is a serious health issue for parents^1^. Previous research has demonstrated the negative impact of maternal depression on infant hospitalization and mortality^2^, duration of breastfeeding^3^, parenting^4^, and child development^5,6^. Compared with maternal depression, paternal depression has not been recognized as a serious health issue within the general population worldwide. However, the negative effects of paternal depression on maternal and child health have been indicated by previous studies. These include an increase in inappropriate parenting behaviour^7^, impaired bonding with infants^8^, less enriching parenting activities^9^, adverse effects on emotional, behavioural, and psychosocial development in child^10,11^, and depression in adolescence^12^. It is also known that paternal depression has the potential to increase father’s risk of suicide^13^ and healthcare costs^14^. According to a recent meta-analysis and systematic review, the estimate for paternal depression in the first year after childbirth is 8.4% [95% Confidence Interval (CI), 7.2–9.6%]^15^. The estimate for maternal depression is slightly higher [11.9%, (95% CI: 11.4%–12.5%)]^16^. The prevalence rates for any anxiety disorder among fathers in the postnatal period ranged from 2.4–18.0%^17^. The prevalence of depressive episodes in fathers in the first year after birth was relatively high, compared with later years^18^.

Previous studies have shown that paternal depression and maternal depression are positively correlated^15,19–22^, and the occurrence of depression in one parent led to an increased risk of the depression in the other^23,24^. In addition, the prevalence of both parents being simultaneously depressed at nine months postpartum was 2.9%, and research has shown that parental behaviour and breastfeeding practice deteriorate when both parents are depressed at the same time^9^. Thus, it is critical to prevent parents from becoming psychologically distressed at the same time to prevent a poor quality of child-rearing practices as well as the possibility of adverse effects such as the children’s subsequent developmental delay^10,11^. However, the evidence is lacking regarding the prevalence and factors associated with individual psychological distress and simultaneous psychological distress in both partners in the postpartum period.

Therefore, the purpose of this study was to describe the prevalence of paternal and maternal psychological distress at both the individual and simultaneous psychological distress in both partners and to explore the factors associated with both parents experiencing simultaneous psychological distress using a nationwide cross-sectional survey in Japan.

## Results

Descriptive information for the participants is presented in Table 1. The mean ages of fathers and mothers were 33.9 years (*SD* = 6.0) and 32.1 years (*SD* = 5.1), respectively. The number of boys was 1,765 (50.2%) and the number of girls was 1,749 (49.8%). The numbers of households with one child and those with two or more children were 1,593 (45.3%), and 1,921 (54.7%), respectively. Households in which parents were the main caregivers of children during daytime were 2,257 (67.4%). Of the fathers, 3,479 (99.3%) were employed and 872 (26.4%) fathers reported working more than 55 h per week. Among the mothers, those who were employed, and those who worked one hour or more per week were 1,545 (44.0%), and 653 (19.6%), respectively.

**Table 1:** Basic characteristics of households, parents, and children (n = 3,514).

			Household		Father		Mother	
			n	%	n	%	n	%
**Household and child characteristics**								
Residential area		Metropolitan	793	22.6				
		City with a population of over 50,000	1,999	56.9				
		City with a population of less than 50,000	722	20.5				
Number of children		1	1,593	45.3				
		≥ 2	1921	54.7				
Twin		Yes	35	1.0				
Age in months of youngest children		0–3 months	734	20.9				
		3–6 months	872	24.8				
		6–9 months	937	26.7				
		9–12 months	971	27.6				
Monthly household expenditure per person^a^ (median, IQR)			5.7	4.0–7.5				
Sex of youngest children		Male	1,765	50.2				
Living with grand parents		With father's parent(s)	450	12.8				
		With mother's parent(s)	111	3.2				
Primary caregiver of children during daytime		Parents only	2,257	67.4				
		Parents and any of other family members and nursery	1,092	32.6				
**Parental characteristics**								
Age (mean, SD)					33.9	6.0	32.1	5.1
Education^b^		Low			1,859	58.5	2,212	69.9
		High			1,320	41.5	952	30.1
Visits hospital regularly		Yes			571	16.3	584	16.7
Visits hospital due to mental health problem		Yes			24	0.7	28	0.8
Number of hours sleeping per day		< 6 h			1,426	40.6	1,803	51.4
		≥ 6 h			2,084	59.4	1,704	48.6
Smoking (everyday or occasional)		Yes			1,407	40.4	188	5.4
Alcohol use (≥ once per week)		Yes			1749	50.2	207	5.9
Employment status		Yes (Undertaking childcare leave)			3,479	99.3	1,545	44.0
Number of work hours per week		< 55 h			2,431	73.6		
		≥ 55 h			872	26.4		
Number of work hours per week		No work hours					2,675	80.4
		≥ 1 h					653	19.6

^a^Monthly household expenditure per person in May, 2016 (10,000 JPY).

^b^Defined graduation from 2-year college, vocational school, or less as low and 4-year college or graduate school as high.

The prevalence of psychological distress at both the individual and couple level in the first year after childbirth is shown in Table 2. The Japanese version of the Kessler Psychological Distress Scale (K6) was used to assess moderate psychological distress (MPD), defined as a score of 9–12, and severe psychological distress (SPD), defined as a score ≥ 13. The prevalence of MPD or SPD (i.e. K6 ≥ 9) during the first year after delivery was 11.0% for fathers and 10.8% for mothers. The prevalence of SPD (i.e. K6 ≥ 13) was 3.7% for fathers and 3.5% for mothers.

**Table 2:** Prevalence of moderate and severe psychological distress among parents by age in months in the first year after birth (n = 3,514).

		Age in months of their youngest children				Overall period (0–12 months)
		0–3 months	3–6 months	6–9 months	9–12 months	
**Fathers**						
MPD or SPD		10.4%	11.0%	11.1%	11.2%	11.0%
SPD		3.3%	3.7%	4.5%	3.3%	3.7%
**Mothers**						
MPD or SPD		11.2%	8.7%	11.7%	11.5%	10.8%
SPD		3.0%	2.9%	4.5%	3.4%	3.5%
**Either a couple**						
MPD or SPD		14.7%	16.1%	13.9%	15.6%	15.1%
SPD		4.0%	5.8%	6.3%	4.5%	5.2%
**Both of couple**						
MPD or SPD		3.4%	1.8%	4.5%	3.6%	3.4%
SPD		0.7%	0.1%	0.3%	0.5%	0.4%

MPD (moderate psychological distress): the prevalence of the K6 score between 9 and 12 points.

SPD (severe psychological distress): the prevalence of the K6 score ≥ 13 points.

MPD or SPD: the prevalence of the K6 score ≥ 9 points.

The prevalence of households in which either parent was assessed at a K6 rating of ≥ 9 (i.e. MPD or SPD) was 15.1% and ≥ 13 (i.e. SPD) was 5.2% in the first year after delivery. The prevalence of households in which both parents were assessed at a K6 rating ≥ 9 was 3.4% and a K6 rating ≥ 13 was 0.4%.

The associations and crude odds ratios (COR) between paternal and maternal MPD or SPD and between paternal and maternal SPD using chi-square analyses are presented in Supplementary Tables 1 and 2. Both analyses showed significant associations between paternal and maternal psychological distress, and the crude odds ratios were 4.84 (95% CI, 3.76–6.22) and 3.66 (95% CI, 2.04–6.58), respectively.

The results of the univariate and multivariate analysis used to explore the associations with both parents experiencing psychological distress at the same period are shown in Table 3. In the univariate analysis, both parents assessed as having MPD or SPD at the same time was significantly associated with the age in months of the youngest child, monthly household expenditure per person, and mothers or fathers who slept less than 6 h per night.

**Table 3:** Crude and adjusted odds ratio between parental psychological distress (both parents scored K6 ≥ 9; MPD or SPD) and potential risk factors such as socio-economic status, health behavior, and working situation among Japanese parents. (n = 3,514).

			Both parents scored K6 ≥ 9 (n = 118)		Either or both parents scored K6 < 9		P	Crude OR		Adjusted OR	
			n	%	n	%		(95%CI)		(95%CI)	
**Household**											
Residential area		Metropolitan	25	3.2	768	96.8	0.889	Ref		–	–
		City with a population of over 50,000	67	3.4	1,932	96.6		1.07	(0.71–1.70)	–	–
		City with a population of less than 50,000	26	3.6	696	96.4		1.15	(0.70–2.01)	–	–
Number of children		1	49	3.1	1,544	96.9	0.398	Ref		Ref	
		≥ 2	69	3.6	1,852	96.4		1.17	(0.83–1.70)	1.30	(0.84–2.01)
Twin^a^		No	118	3.4	3,361	96.6	0.631	–	–	–	–
		Yes	0	0	35	100.0		–	–	–	–
Month age of youngest children		0–6 months	41	2.6	1,565	97.4	0.015	Ref		Ref	
		6–12 months	77	4.0	1,831	96.0		1.61	(1.09–2.36)	1.58	(1.02–2.45)
Sex of youngest children		Male	62	3.5	1,703	96.5	0.609	Ref		Ref	
		Female	56	3.2	1,693	96.8		0.91	(0.65–1.31)	0.96	(0.64–1.45)
Monthly household expenditure per person		Lower half	41	2.4	1,681	97.6	0.002	Ref		Ref	
		Upper half	77	4.3	1,715	95.7		1.84	(1.25–2.71)	2.09	(1.33–3.28)
Main caregiver of children during daytime		Parents and any of other family members and nursery	47	4.3	1,045	95.7		Ref		Ref	
		Parents only	69	3.1	2,188	96.9	0.064	0.70	(0.50–1.02)	0.74	(0.46–1.20)
**Father**											
Age		< 30	32	4.0	774	96.0	0.272	Ref		Ref	
		≥ 30	86	3.2	2,622	96.8		0.79	(0.53–1.20)	0.90	(0.57–1.40)
Education		Low	66	3.6	1,793	96.4	0.572	Ref		Ref	
		High	42	3.2	1,278	96.8		0.89	(0.62–1.32)	0.90	(0.57–1.40)
Under treatment other than mental illness		No	99	3.3	2,864	96.7	0.816	Ref		Ref	
		Yes	19	3.5	518	96.5		1.06	(0.65–1.73)	1.05	(0.60–1.83)
Smoking		No	61	2.9	2,011	97.1	0.077	Ref		Ref	
		Yes	57	4.1	1,350	95.9		1.39	(0.98–2.01)	1.54	(0.99–2.39)
Alcohol use (≥ once per week)		No	65	3.7	1,670	96.3	0.205	Ref		Ref	
		Yes	52	3.0	1,697	97.0		0.79	(0.56–1.14)	0.72	(0.47–1.10)
Number of hours slept each night		≥ 6 h	59	2.8	2,025	97.2	0.035	Ref		Ref	
		< 6 h	59	4.1	1,367	95.9		1.48	(1.04–2.14)	1.28	(0.84–1.95)
Number of work hours per week		< 55 h	76	3.1	2,355	96.9	0.087	Ref		Ref	
		≥ 55 h	38	4.4	834	95.6		1.41	(0.96–2.10)	1.61	(1.05–2.49)
**Mother**											
Age		< 30	36	3.3	1,059	96.7	0.876	Ref		–	–
		≥ 30	82	3.4	2,337	96.6		1.03	(0.69–1.54)	–	–
Education		Low	82	3.7	2,130	96.3	0.166	Ref		–	–
		High	26	2.7	926	97.3		0.73	(0.48–1.14)	–	–
Under treatment other than mental illness		No	97	3.3	2,856	96.7	0.809	Ref		Ref	
		Yes	19	3.5	526	96.5		1.06	(0.73–1.89)	1.16	(0.67–2.00)
Smoking		No	113	3.4	3,187	96.6	0.573	Ref		Ref	
		Yes	5	2.7	183	97.3		0.77	(0.33–1.91)	0.51	(0.18–1.48)
Alcohol use (≥ once per week)		No	109	3.3	3,176	96.7	0.426	Ref		Ref	
		Yes	9	4.3	198	95.7		1.32	(0.68–2.65)	1.27	(0.53–3.06)
Number of hours slept each night		≥ 6 h	44	2.6	1,660	97.4	0.020	Ref		Ref	
		< 6 h	72	4.0	1,731	96.0		1.57	(1.10–2.30)	1.81	(1.17–2.79)
Number of work hours per week		No work hours	82	3.1	2,593	96.9	0.117	Ref		Ref	
		≥ 1 h	28	4.3	625	95.7		1.42	(0.93–2.19)	1.13	(0.64–1.95)

2,773 couples were included in the multivariate analysis.

In multivariate logistic regression including 2,773 (78.9%) of the sample, both parents being assessed as having MPD or SPD at the same time was significantly associated with fathers who worked 55 h or more per week (adjusted odds ratio (AOR), 1.61; 95% CI, 1.05–2.49), mothers who slept less than 6 h per night (AOR, 1.81; 95% CI, 1.17–2.79), children aged 6–12 months (vs. children aged 0–6 months; AOR, 1.58; 95% CI, 1.02–2.45), and high household expenditure per person (lower half vs. upper half: AOR, 2.09; 95% CI, 1.33–3.28). No other variables were found to be significantly associated with both parents reporting MPD or SPD at the same time.

For a sensitivity analysis, fathers who were assessed as having MPD or SPD were significantly more likely in the multivariate analysis (Supplementary Table 3) to be have slept less than 6 h per night, work 55 h or more per week, and have a high monthly household expenditure per person. Mothers who were assessed as having MPD or SPD were significantly more likely in the multivariate analysis (Supplementary Table 4) to be mothers who had husbands who smoked, had lower alcohol consumption, had a child aged 6–12 months, and being mothers who slept less than 6 h per night.

## Discussion

This is the first study to describe the trends in the prevalence of psychological distress in the first year after delivery and their associated factors at both at the individual level and simultaneous psychological distress in both partners. We identified factors associated with both parents having MPD or SPD at the same time during the first year postpartum using data from a nationwide population-based survey. Overall, we found that approximately 11% of fathers and mothers were at risk for either MPD or SPD in the first year after the birth of their child based on ratings on the K6. We also found that the proportion of households in which either or both parents were identified as having MPD was 15.1% or SPD was 3.4%. When one parent developed MPD or SPD, the odds ratio of the partner developing MPD or SPD was 4.84 times higher and developing SPD was 3.66 times higher compared with those partners without distress.

The response rate of the Comprehensive Survey of Living Conditions in Japan (CSLC) in 2016 was 77.5%. Most of the non-respondents of the CSLC were young and living in single households, especially those who were living in urban areas, when compared to the distribution of the National Census^25^. Thus, the representativeness of the population of this study compared to the census population is high.

In comparison with the prevalence of paternal, maternal, and both parents’ depression at nine months postpartum that was reported in a previous study conducted in the United States, which were 7.4%, 11.5%, and 2.9%, respectively^9^, the prevalence of fathers, mothers, and both of couple having MPD or SPD between 9–12 months postpartum in this study was higher (11.2%, 11.5%, and 3.6%, respectively). The prevalence of mothers was almost same^9^. The prevalence of psychological distress for fathers in this study was higher than the prevalence of paternal depression reported in a previous meta-analysis^15^, but the prevalence of psychological distress for mothers in this study was similar to the prevalence of maternal depression reported in a previous systematic review^16^. We found that the prevalence of MPD or SPD in this period might be different according to the age in months of the youngest child because the prevalence of 0–6 months postpartum for both parents and mothers was lower than those parents whose youngest child was 6–12 months postpartum.

To the best of our knowledge, this is also the first study using a national survey to explore the factors associated with both parents experiencing psychological distress in the same period in the first year after birth. Fathers who worked 55 h or more per week, mothers who slept less than 6 h per night, the age in months of the youngest child, and high household expenditure per person, were associated with both parents having MPD or SPD at the same time. The negative effects of long work hours and working weekends on the mental health status of both parents have previously been reported^26–28^. Our results have shown that 26.4% of fathers worked 55 h or more per week in the first year after delivery and that long work hours among fathers may raise the possibility of psychological distress among fathers as well as mothers. There are a number of systematic reviews that have examined poor and interrupted sleep and its negative effects on mental health in the postnatal period^29–31^, and parents who are raising young children need to have time for housework, childcare, and rest at home. In particular, there are increased time demands related to childcare, such as feeding, changing diapers, and getting children to sleep during the initial years after birth. If the father works long hours, the primary responsibility for most of the housework and childcare falls upon the mother, who may not receive enough support from her partner. As a result, both parents may be exhausted by the fathers’ long working hours. The promotion of work-life balance is an urgent issue for parents with young children to improve their quality of life and mental health.

Although a significant association between higher monthly household expenditure per person and both parents experiencing psychological distress in the same time period was observed, we cannot conclude a causal relationship. The relationship between mental health problems and economic burdens is an issue that has been extensively examined at the global, national, household, and individual levels^32–34^. In addition to the increase in the healthcare cost in society, factors that contribute to mental health problems include decreases in earnings, increases in household expenditures, and the unpaid costs for informal caregivers for those with mental illnesses. In this study, we speculate that the increase in the household expenditure is not the cause of psychological distress but the result. Further longitudinal studies are necessary to show the effect of psychological distress on household expenditure.

Even when one parent experiences poor mental health, the quality of child-rearing in the home deteriorates^3,7,9,35^, so the adverse effect on the child’s environment may be even greater if both parents are psychologically distressed. From the perspective of child development and parental quality of life, an environment in which both parents experience psychological distress may be a critical situation that should be addressed as soon as possible. Unintentionally, parental psychological distress may affect the level of care given to children, even leading to neglect. Neglect during early childhood is known to contribute to poor outcomes in later life, such as antisocial behaviour in adolescent boys and depression in adults^36,37^.

### Limitations

The current study has several limitations that should be considered. First, the K6 in the CSLC was assessed not through a structured interview but by a self-administered questionnaire. This scale, when previously used and developed in the United States^38^, and then validated in Japan^39^, was administered using a structured interview. Second, the identification of parents and children was made using a parent/child identification variable included in the CSLC dataset. Therefore, we are not entirely certain of the biological relationship between the parents and children as parent–child relationships, foster parents, and other familial arrangements that were included in the original data collection. Third, reverse causation should be considered in this study because CSLC employed a cross-sectional design; however, the negative effects of long work hours^26–28^ and poor sleep^29–31^ on mental health have been well established in previous research. In addition, age cannot suddenly change according to an event because the age of a child increases continuously and at a regular interval. Fourth, the effects of data sparse bias in the multivariate analysis should be considered because of the large number of covariates compared to the number of outcome events. However, there were no noticeable differences of the direction or value of the odds ratios between the univariate and multivariate analyses. Finally, the effects of psychological history, including mental health status and psychiatric consultation in the prenatal period and before the pregnancy, which is well known as an important risk factor for mental health status in the postnatal period of both mothers and fathers^40,41^, were not adjusted for in the multivariate analysis as the variables were limited in the CSLC. Despite these limitations, this study, which used a nationwide population-based dataset and included couples, identified several important findings.

## Conclusion

The prevalence for either or both parents experiencing MPD or SPD in the first year after birth was 15.1% and 3.4%, respectively. The condition where both parents reported having MPD or SPD at the same time was associated with fathers worked 55 h or more per week, reduced hours of sleep, age in months of the youngest child, and high household expenditures. To prevent parental psychological distress, mental health assessments during the postpartum period should be promoted among both mothers and fathers. Especially, the workplace may have an important role in assessment and support of psychological distress among fathers because there are few other ways to outreach them during this period. Along with enhancing the quality of health service, improving the promotion of work-life balance is another urgent issue for parents with young children to promote parental mental health.

## Methods

### Study population

We analysed the data from the Comprehensive Survey of Living Conditions in Japan (CSLC), which is a repeated national cross-sectional survey conducted by the Ministry of Health, Labour, and Welfare of Japan (MHLW). A summary of the CSLC is published each year^42^. The CSLC applied a stratified random-sampling method based on enumeration districts from the annual census. In the 2016 survey, 5,410 enumeration districts were selected randomly, and all of the members of the 289,470 households within the selected districts were recruited for participation. Individuals who were hospitalized, institutionalized, or on long-term business trips were excluded from the CSLC. Valid responses were collected from 224,208 households (response rate: 77.5%), which comprised 568,426 members. The survey was implemented on June 2, 2016. The data is one of the government survey data with limited accessibility.

**Figure 1:**
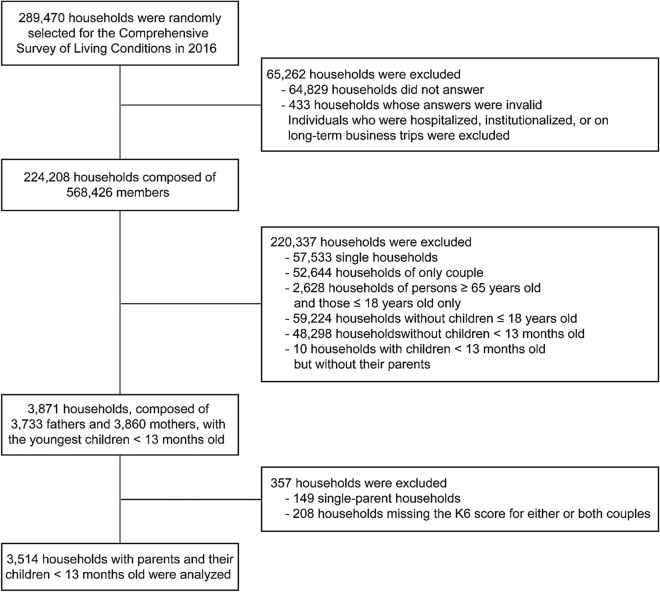
Flow chart of data extraction.

The inclusion criteria in this study were (1) being a couple who participated in the CSLC in 2016; (2) either or both parents were 65 years or younger, and (3) being a couple who had a child < 13 months old. The exclusion criterion was having missing values on the K6 scores for either or both parents. The flow chart of data extraction is shown in Fig. 1. Out of the 224,208 households responding to the CSLC, we extracted 3,871 households with at least one child under the age of 12 months for analysis in this study. There were 38 households with two children under one year old. Of these 38 households, 34 households had twins, and the remaining four households had non-twin siblings. In the case of non-twin siblings, only data from the younger child were included. Out of the 3,871 households, 149 households which had either no father or mother, and 208 households that did not have a K6 score for either or both parents were excluded from this study. In total, 3,514 households, composed of 3,514 fathers and 3,514 mothers, met the above criteria from the original CSLC data set and were included in the final analysis.

### Measurement

The individual and household data were collected via a self-administered questionnaire as part of the CSLC. A data collector distributed and collected the questionnaires during visits to participants’ homes. Psychological distress was assessed using the Japanese version of the Kessler Psychological Distress Scale (K6) in the CSLC questionnaire. The K6 consists of six questions that examine the frequency during the last 30 days of the following items: (1) nervousness, (2) hopelessness, (3) restlessness or fidgetiness, (4) being so depressed that nothing could cheer you up, (5) feeling that everything is an effort, and (6) worthlessness. The responses for each question range from 0: *None of the Time* to 4: *All of the Time* with total scores ranging from 0 to 24 points. The K6 has been validated against the classifications for anxiety and mood disorder of the American Psychiatric Association’s *Diagnostic and Statistical Manual of Mental Health Disorders, 4th edition*, in previous studies via structured interviews^38,39^.

The optimal cut-off scores of the Japanese version of the K6 has been examined. The K6 score ≥ 13 is often used to indicate severe or serious psychological distress^43–46^. The performance of the Japanese version of the K6 was examined using the areas under the receiver operator characteristics curves (AUCs) and stratum-specific likelihood ratios (SSLRs)^39^. The AUC was excellent with a high value of 0.94 (95% CI, 0.88–0.99) and the SSLRs for a score of between 6–8 points, 9–13 points, 14–24 points on K6 were 4.9 (95% CI, 1.7–11.2), 16 (95% CI, 6.1–34.0), and 110 (95% CI, 11–400), respectively^39^. A likelihood ratio greater than 10 is considered to be an informative criterion in the diagnostic process for a disease^47,48^. These results showed that a K6 score ≥ 9 is one of the optimal cut-off points, although some previous studies have adopted a K6 score ≥ 5 as a cut-off point to define moderate psychological distress^46,49,50^. The same cut-off scores on the K6 are typically adopted for Japanese men and women. Therefore, in the current study, for both fathers and mothers, we used a score between 9 and 12 to indicate moderate psychological distress (MPD), and a score of greater than or equal to 13 was used to indicate severe psychological distress (SPD).

Age in months of the youngest child was calculated using their birth year and month on the date of survey implementation, which was June 2, 2016. The date of their birth was not surveyed in the CSLC. For example, in the case of children born in March 2016 and in September 2015, their age was classified as two months old and eight months old, respectively. As an exception, a child born in either May or June 2016 was classified as being less than one month of age because of proximity to the date of the CSLC. The age in month of the youngest child was divided into two periods: birth to 6 months, 0 days postpartum (0–6 months); and6 months, 1 day to 12 months, 0 days postpartum (6–12 months). In addition, birth to 12 months, 0 days was defined as the overall study period (0 to 12 months). This period was used to consider results presented in the latest meta-analysis regarding paternal depression^15^.

Participants were asked to consider their monthly household expenditure per person and employment status in the past month. The number of work hours per week was provided for the period between16th May to 22nd May. The average number of hours slept per night in May, 2016 was assessed. Different categorizations of weekly work hours were used for fathers and mothers in this study. In Japan, work hours are defined in the Labor Standards Law as being 40 h or less per week, and overtime hours that are up to 15 h per week are allowed by the specific labor-management agreement pursuant to Article 36 of the Labor Standard Act. Therefore, working 55 or more hours in a week was defined as working inappropriately long work hours for fathers in this study. In contrast, most women utilize childcare leave in the first year after delivery. Thus, weekly work hours for mothers were dichotomized based on whether they worked one hour or more per week.

### Data analysis

We measured the means and frequencies of the socioeconomic status, health behaviours, and working environments in the household for both fathers and mothers. The proportions of households in which either one or both parents were assessed as having MPD and SPD were also reported. The crude odds ratio (COR) with 95% CI for the association between paternal and maternal psychological distress (K6 ≥ 9 and K6 ≥ 13) was calculated using univariate logistic regression.

The crude odds ratios with 95% CI for the association between both parents scoring K6 ≥ 9 and K6 ≥ 13 were calculated using a univariate analysis. The parity, monthly household expenditure, main caregiver of children during daytime, children’s characteristics such as age in months and sex, paternal characteristics such as age, education, health condition, smoking, drinking, number of hours slept, and number of work hours, and maternal characteristics such as health condition, smoking, drinking, number of hours slept, and number of work hours, were analysed using univariate and multivariate logistic regression with complete-case analysis in each analysis. Parental, paternal, and maternal psychological distress were set as the dependent variables. In the multivariate analysis, 2,773 couples were included without any missing variables. As a sensitivity analysis, the COR and AOR with 95% CI was calculated to examine the association between these factors, paternal, and maternal psychological distress factors separately.

The alpha level was set at 5%. No imputations were completed for missing data in this study. All statistical analyses were performed with IBM SPSS statistics version 19.0 (IBM, Armonk, NY).

### Ethical considerations

The use of de-identified individual-level data from the CSLC for scientific research was approved by the MHLW through the official application procedure under Article 33 of the Statistics Act (March 1, 2018). The informed consent was waived in the CSLC because this fundamental statistical survey was conducted to be based on the Statistics Act. We also did not obtain the consent in this study because we performed only secondary data analysis using the national statistics. This study was approved by the Japanese National Center for Child Health and Development ethics committee (No. 1533). This study was conducted to be based on the Ethical Guidelines for Medical and Health Research Involving Human Subjects in Japan.

## Supplementary information

Supplementary Information.
